# The cause and effect of gut microbiota in development of inflammatory disorders of the breast

**DOI:** 10.1186/s40001-023-01281-6

**Published:** 2023-09-07

**Authors:** Yibo Gu, Muye Hou, Jinyu Chu, Li Wan, Muyi Yang, Jiemiao Shen, Minghui Ji

**Affiliations:** 1 School of Nursing, Nanjing Medical University, Nanjing, 211166 People’s Republic of China; 2 Department of Obstetrics, Nanjing Hospital Affiliated to Nanjing Medical University, Nanjing, 210006 People’s Republic of China

**Keywords:** Gut microbiota, Inflammatory disorders of the breast, Mendelian randomization study, Causal reasoning

## Abstract

**Background:**

Inflammatory disorders of the breast (IDB) damages the interests of women and children and hinders the progress of global health seriously. Several studies had offered clues between gut microbiota (GM) and inflammatory disorders of the breast (IDB). The gut–mammary gland axis also implied a possible contribution of the GM to IDB. However, the causality between them is still elusive.

**Methods:**

The data of two-sample Mendelian randomization (MR) study related to the composition of GM (n = 18,340) and IDB (n = 177,446) were accessed from openly available genome-wide association studies (GWAS) database. As the major analytical method, inverse variance weighted (IVW) was introduced and several sensitive analytical methods were conducted to verify results.

**Results:**

Inverse variance weighted revealed *Eubacterium rectale group* (OR = 1.87, 95% CI: 1.02–3.43, *p* = 4.20E−02), *Olsenella* (OR = 1.29, 95% CI: 1.02–1.64, *p* = 3.30E−02), *Ruminiclostridium-6* (OR = 1.53, 95% CI: 1.08–2.14, *p* = 1.60E−02) had an anti-protective effect on IDB. *Peptococcus* (OR = 0.75, 95% CI: 0.60–0.94, *p* = 1.30E−02) had a protective effect on IDB. The results were credible through a series of test.

**Conclusions:**

We revealed causality between IDB and GM taxa, exactly including *Ruminiclostridium-6*, *Eubacterium rectale group*, *Olsenella* and *Peptococcus*. These genera may become novel biomarkers and supply new viewpoint for probiotic treatment. However, these findings warrant further test owing to the insufficient evidences.

**Supplementary Information:**

The online version contains supplementary material available at 10.1186/s40001-023-01281-6.

## Background

Inflammatory disorders of the breast (IDB) could be categorized into lactational mastitis (LM) and non-lactational mastitis (NLM) according to the time of occurrence [1]. The reported incidence has shown the IDB ranges from 3 to 33% of women in lactation period, and less than 10% in non-lactating ones [2, 3]. Whether LM or NLM, to resist distinct clinical manifestations of localized and associated systemic symptoms, women commonly adopt antibiotic therapy [4, 5]. Delayed treatment may cause severe outcomes such as sepsis for LM and breast fistula for NLM. Breast abscess is also a potential complication for IDB [6]. Due to the long treatment duration, ineffective adopting antibiotic and easy recurrence, the treatment of NLM faces tremendous challenge [7, 8], which may result in considerable economic burden and psychological distress in women. In addition, breastfeeding is utmost important and is considered as the origin of life. The beginning and development of LM may cause premature cessation of breastfeeding, suffering to both mothers and children [9]. Despite routine treatment including antibiotic has been used extensively, the effectiveness and security of antibiotic therapy has not been confirmed yet [8, 10, 11]. Thus, it is crucial to clarify the etiology of IDB and to prevent the occurrence of IDB from its root causes. However, tangible etiology concerning IDB remains unclear due to research deficiency [12, 13]. Therefore, considering the benefits of health and current treatments are not all effective, it is imperative to seek the etiology of IDB.

The GM, familiar with the "second genome of the human", is tightly linked to our benefits and disorders [14]. Due to the presence of gut–mammary gland axis, gut dysbiosis may contribute to the occurrence and development of breast disorders [15, 16]. Animal studies have proven disturbance of GM and related metabolites induced the development of IDB in mice [17], and feces microbiota transplantation (FMT) could reverse adverse effects [18]. Microbiota-depleted mice developed IDB symptoms when were transplanted with the GM from unhealthy cows with IDB [19]. Nevertheless, the evidence of randomized controlled trials (RCTs) between IDB and GM is scanty and has not been fully evaluated [20]. In addition, observational studies of GM and IDB are vulnerable to external factors such as genotyping of gut microbial community, dietary appetite, mood and life mode [21, 22]. It is unknown whether the specific taxa of GM cause IDB or not. Therefore, it is urgent to confirm causality of GM on IDB and to understand which microbiota taxa developing IDB.

Due to limitations of medical ethics and high costs, some RCTs are difficult to carry out in practical work [23]. MR study was introduced to exploit in the inference of epidemiological causes. Based on Mendel's Laws of Inheritance, MR could progress causal inference among exposure and outcome [24]. Mounting MR analysis has been introduced to confirm the causality between GM and disorders, by way of example, cancers [25], cardiovascular diseases [26] and depressive disorder [27]. In this study, MiBioGen and FinnGen consortiums, two large GWAS databases, were employed for statistical analysis. A two-sample MR design was conducted to verify causality and to provide a theoretical foundation for the etiology and biomarker of IDB.

## Methods

### The assumptions and study design of MR

**Fig. 1:**
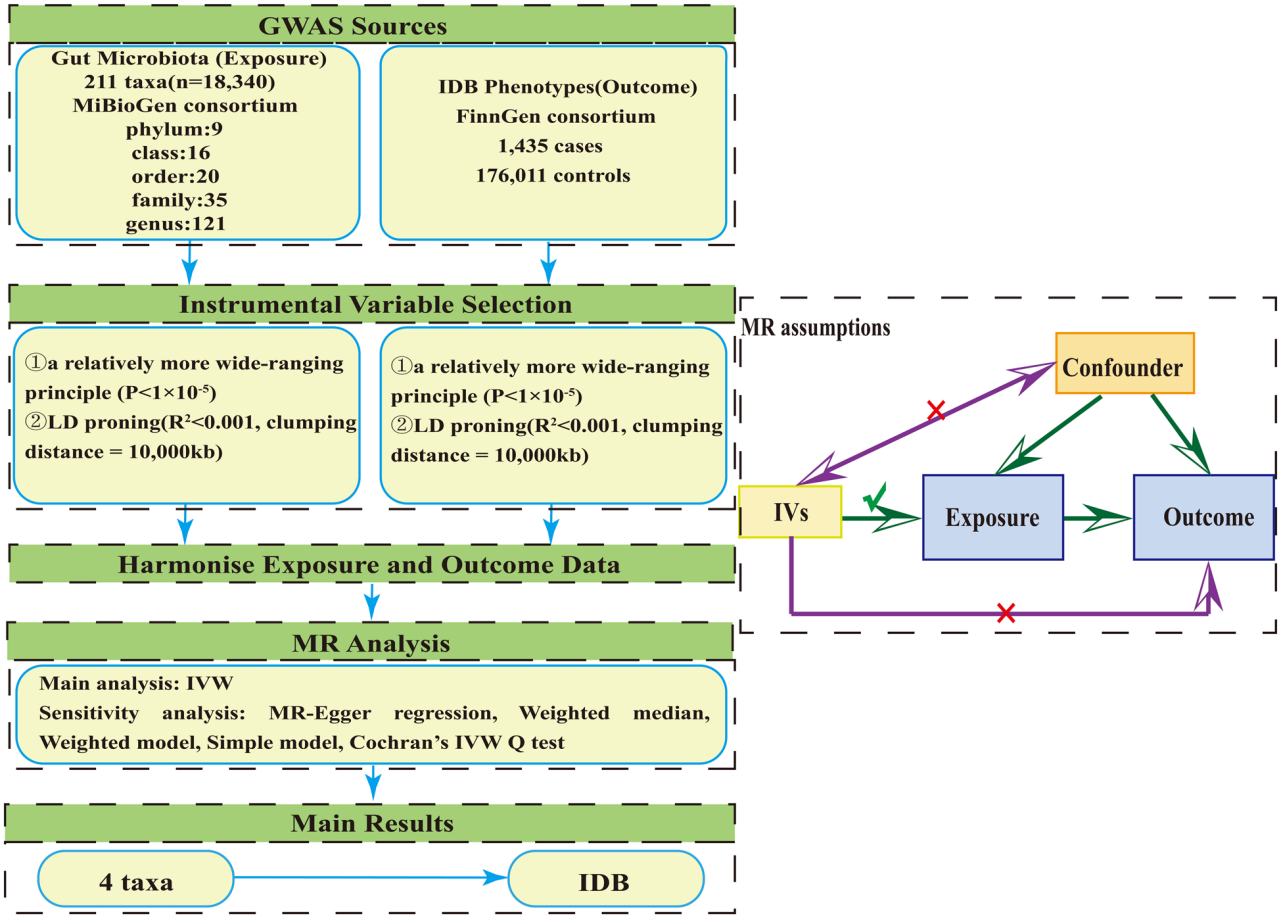
Study design and MR assumptions

The diagrammatic sketch of this research is illustrated in Fig. 1. Briefly, the exposure is the GM, whereas the outcome is IDB. Moreover, reliable results are based on the following 3 assumptions of MR analysis [28]: (1) the closely relationship between the instrumental variables (IVs) and exposure should be a must; (2) IVs should be independent, ensuring no relation with confounding factors; (3) IVs influenced outcome through exposure rather than other factors.

### Data sources

This research related summary-level data were downloaded from openly GWAS database. In detail, the GWAS data on GM originated from MiBioGen consortium [29–31] and the GWAS data relating IDB were mainly conducted by the Finngen consortium [32, 33]. Ethical approval and consent of GWAS database were achieved, and the summary-level data were publicly available and could be used.

MiBioGen consortium included 24 large cohorts (18,340 participants) from most European countries. 16S rRNA sequencing was used to explore composition of microbial communities and its classification via microbial classification standards [34]. 122,110 variant sites from 211 taxa were obtain in microbiota-GWAS. Owing to 12 unknown genera and 3 unknown families, a total of 196 taxa were included for analysis in the end. In our study, we selected IVs from genus to phylum level of GM taxa. For more detailed information, please refer to original articles [29]. According to the International Statistical Classification of Diseases and Related Health Problems 10th Revision (ICD-10), this phenotype is “inflammatory disorders of the breast” (ICD-10 code N61). IDB is defined as the inflammation of breast tissue during lactation or postpartum due to an obstructed duct or infection. IDB can also occur in non-breastfeeding women, and rarely in men. We use this phenotype for the following reasons: firstly, enough types of relating diseases: this phenotype excludes neonatal infective mastitis, includes (1) acute, chronic and nonpuerperal abscess of areola and breast; (2) carbuncle of breast; (3) acute, subacute and nonpuerperal mastitis. Secondly, profound impacts of relating diseases: we have ploughed through relating documents that whatever disease which leading to the inflammation of breast tissue may result in the interruption of lactation and have impact on mother and children health [35, 36]. Therefore, once women develop IDB, this adverse state could inevitably affect women themselves and if women were in lactation period, it could bring breastfeeding crisis. Thirdly, this consortium was large enough to explore the causality between GM and IDB. Above all, we introduced this consortium. A total of 177,446 participants were involved in this GWAS. Among this GWAS, it recruited 177,446 female subjects and divided into 1435 cases and 176,011 controls. A series of corrections have been made during the performance [32].

### Instrumental variables (IVs)

The selection criteria of IVs were following: (1) previous articles were referred to formulate a relatively more wide-ranging principle (*p* < 1 × 10^–5^) [37, 38]. Therefore, *p* < 1 × 10^–5^ was performed because of the less eligible IVs (*p* < 5 × 10^–8^) [39, 40]. (2) 1000 Genomes project European samples data were referenced to compute the linkage disequilibrium (LD) (*R*^2^ < 0.001, clumping distance = 10,000 kb) between the single nucleotide polymorphisms (SNPs), these SNPs with the lowest *P*-values would be eventually reserved. (3) Under the presence of palindromic SNPs circumstances, we used allele frequencies to infer positive strand alleles. (4) During the comparing process, we checked the alleles against Genome Reference Consortium Human Build 38 and removed indeterminate and duplicated SNPs.

### Statistical analysis

R software (Version 4.1.0) and R package TwosampleMR (Version 0.56) were performed to this statistical analysis. We carried out *p* < 0.05, a threshold of statistical significance, as a potential causal effect.

During this statistical analysis, several methods were performed to determine the causality between GM and IDB. IVW is a meta-analysis method used by MR to analyze the effects of multiple SNPs at multiple loci. The application premise of IVW is that all SNPs are valid IVs and completely independent of each other. Based on this, the unbiased of IVW results would be presented [41]. MR-Egger regression does not force the regression line to pass through the origin, allowing for targeted gene pleiotropy in the included instrumental variables. When the regression intercept is not zero and *p* for intercept < 0.05, it indicates the existence of gene pleiotropy [42]. The weighted median is the median of the distribution function obtained after all individual SNP effect size are sorted by weight. When at least 50% of the information comes from effective instrumental variables, weighted median can obtain robust estimates [43]. MR-PRESSO is a method of evaluating horizontal polymorphism using whole genome aggregated association statistical data. MR-PRESSO has three components, including MR-PRESSO overall test, MR-PRESSO outlier test and MR-PRESSO distortion test. Specific SNPs can be excluded by excluding outlier to obtain an estimate closer to the true value [44]. The weighted model and simple model also used to evaluate the effectiveness and correctness of MR calculations [45]. The simple mode takes the largest cluster of SNPs’ causal estimation, and the weighted mode assigns the weights to each SNP [46]. Finally, Cochran's Q statistic was applied to detect heterogeneity. If the Cochran's Q statistic test has statistical significance, it proves that the results were significant heterogeneity.

The leave-one-out method refers to omitting each SNP in turn, calculating the meta effect of the remaining SNPs, and observing whether the results have changed after removing each SNP. If the results change significantly after removing a certain SNP, it indicates that the potential heterogeneous SNPs have a significant impact on the results [47].

The scatter plot is a plot where the effect of the same SNP on exposure is placed on the horizontal axis, the effect on outcome is placed on the vertical axis, and the slope of the plot represents the causal effect of exposure factors on outcomes. It could visualize the causal effect of exposure on outcomes estimated under different parameter estimation methods [48].

To avoid weak instrument bias, the robustness of IVs could be assessed through *F*-statistic. We adopt formula *F* = $\frac{{R}^{2}\times (N-2)}{(1-{R}^{2})}$ to calculate *F*-statistic. Among which, we could use *R*^*2*^ to represent the degree of exposure explained by IVs with the formula *R*^*2*^ = $\frac{(2\times EAF\times (1 - EAF)\times {beta}^{2})}{(2\times EAF\times (1 - EAF) \times {beta}^{2}) + (2\times EAF\times (1 - EAF)\times N\times SE{(beta)}^{2}},$ where EAF represents the effect allele frequency, beta represents the effect estimate of the genetic variant in the exposure GWAS, SE(beta) represents the standard error of the beta and *N* represents sample size [46, 49, 50]. In general, when the corresponding *F*-statistic was > 10, significant weak instrumental bias could be reduced [51].

In the reverse MR analysis, the exposure is the IDB, whereas the outcome is GM. We selected IVs for each IDB phenotypes by using a much stricter threshold, where the significant threshold (*p* < 5 × 10^–8^) [52, 53]. Additionally, the phenotypes, methods and other settings were consistent with those of forward MR. Under the significant threshold (*p* < 5 × 10^–8^), no eligible SNP as IV was selected. A reverse MR analysis was not conducted at last owing to lack of SNPs (related to IDB) satisfying the assumption of the MR study.

## Results

### Selection of IVs

Based on the previous selection criteria of IVs (*p* < 1 × 10^–5^), a total of 2370 SNPs were anchored as IVs related to bacterial taxa from phylum to genus for IDB. For further information, Additional file 1: Table S1 is provided for reference.

### Causal effect of GM on IDB

As shown in Table 1, seven bacterial genera including *Eubacterium rectale group*, *Bifidobacterium*, *Olsenella*, *Peptococcus*, *Prevotella7*, *Ruminiclostridium-6, RuminococcaceaeUCG003* were found to be associated with IDB in at least one MR method. MR methods found no relevance between bacterial taxa from phylum to family for IDB and detailed results are shown in Additional file 1: Table S2. Among seven bacterial genera, *Eubacterium rectale group, Olsenella, Peptococcus* and *Ruminiclostridium-6* were supported by IVW analysis. Specifically, *Eubacterium rectale group* (OR = 1.87, 95% CI: 1.02–3.43, *p* = 4.20E−02), *Olsenella* (OR = 1.29, 95% CI: 1.02–1.64, *p* = 3.30E−02), *Ruminiclostridium-6* (OR = 1.53, 95% CI: 1.08–2.14, *p* = 1.60E−02) had an anti-protective effect on IDB. *Peptococcus* (OR = 0.75, 95% CI: 0.60–0.94, *p* = 1.30E−02) had a protective effect on IDB. In addition, the *F*-statistics of seven bacterial genera selected at least one MR method were all above 10, eliminating the possibility of weak instrument bias (more detailed results are shown in Additional file 1: Table S3).

**Table 1:** MR estimates for the association between gut microbiota and IDB

Exposure	Method	No. of SNP	*F*-statistic	OR	95%CI	*p-*value
*Eubacterium rectale group*	MR Egger	7	213.61	2.18	0.22–21.47	0.53
	Weighted median	7		1.48	0.72–3.06	0.28
	IVW	7		1.87	1.02–3.43	4.20E−02
	Simple mode	7		1.11	0.39–3.19	0.85
	Weighted mode	7		1.22	0.47–3.16	0.7
*Bifidobacterium*	MR Egger	19	611.71	0.38	0.17–0.83	2.70E−02
	Weighted median	19		0.95	0.66–1.37	0.79
	IVW	19		0.98	0.71–1.34	0.88
	Simple mode	19		1.24	0.65–2.37	0.52
	Weighted mode	19		1.02	0.63–1.65	0.94
*Olsenella*	MR Egger	9	191.85	1.36	0.48–3.85	0.58
	Weighted median	9		1.34	0.99–1.81	0.06
	IVW	9		1.29	1.02–1.64	3.30E−02
	Simple mode	9		1.48	0.93–2.37	0.14
	Weighted mode	9		1.49	0.93–2.38	0.14
*Peptococcus*	MR Egger	13	387.2	0.70	0.26–1.84	0.48
	Weighted median	13		0.81	0.59–1.11	0.19
	IVW	13		0.75	0.60–0.94	1.30E−02
	Simple mode	13		0.81	0.49–1.36	0.45
	Weighted mode	13		0.83	0.51–1.34	0.45
*Prevotella7*	MR Egger	11	249.45	1.02	0.18–5.71	0.98
	Weighted median	11		1.38	1.01–1.89	4.60E−02
	IVW	11		1.17	0.89–1.56	0.26
	Simple mode	11		1.54	0.83–2.85	0.2
	Weighted mode	11		1.52	0.82–2.82	0.22
*Ruminiclostridium-6*	MR Egger	14	314.03	1.27	0.54–2.98	0.59
	Weighted median	14		1.62	1.00–2.65	0.05
	IVW	14		1.53	1.08–2.17	1.60E−02
	Simple mode	14		1.58	0.74–3.36	0.25
	Weighted mode	14		1.63	0.86–3.10	0.16
*Ruminococcaceae UCG003*	MR Egger	10	268.82	2.87	0.78–10.57	0.15
	Weighted median	10		1.79	1.04–3.07	3.40E−02
	IVW	10		1.40	0.92–2.15	0.12
	Simple mode	10		2.04	0.89–4.65	0.13
	Weighted mode	10		2.08	0.94–4.61	0.11

*IDB* inflammatory disorders of the breast, *GM* gut microbiota, *SNP* single nucleotide polymorphism, *OR* odds ratio, *CI* confidence interval, *IVW* inverse variance weighted, *MR* Mendelian randomization

### Sensitivity analysis

As displayed in Additional file 1: Table S4, sensitivity analysis was employed to identify the pleiotropy and heterogeneity. The results obtained by MR-Egger regression were as follows: *Eubacterium rectale group* (*p* = 0.90), *Olsenella* (*p* = 0.93), *Peptococcus* (*p* = 0.88), *Ruminiclostridium-6* (*p* = 0.65), *Prevotella7* (*p* = 0.87) and *RuminococcaceaeUCG003* (*p* = 0.29), these six bacterial genera showed no horizontal pleiotropy. However, *Bifidobacterium* (*p* = 0.02) was removed due to the existence of pleiotropy (Table 2). Meanwhile, Cochran’s IVW Q test suggested *Eubacterium rectale group* (IVW: *p* = 0.23; MR Egger: *p* = 0.15), *Olsenella* (IVW: *p* = 0.87; MR Egger: *p* = 0.80), *Peptococcus* (IVW: *p* = 0.92; MR Egger: *p* = 0.88), *Ruminiclostridium-6* (IVW: *p* = 0.76; MR Egger: *p* = 0.71) and *RuminococcaceaeUCG003* (IVW: *p* = 0.28; MR Egger: *p* = 0.31) had no significant heterogeneity except *Prevotella7* (IVW: *p* = 0.04; MR Egger: *p* = 0.03) (Table 2). Interestingly, although no significant pleiotropy and heterogeneity has been founded in *RuminococcaceaeUCG003*, *RuminococcaceaeUCG003* was still filtered out under the IVW results (*p* = 0.12).

**Table 2:** Sensitivity analysis between gut microbiota and IDB

Exposure	Method	*Q*	*Q*_*pval*	MR Egger intercept	MR Egger *pval*	MR PRESSO *pval*
*Eubacterium rectale group*	MR Egger	8.04	0.15	− 0.01	0.90	0.27
	IVW	8.07	0.23			
*Bifidobacterium*	MR Egger	19.68	0.29	0.08	0.02	0.07
	IVW	27.05	0.08			
*Olsenella*	MR Egger	3.84	0.80	− 0.01	0.93	0.87
	IVW	3.84	0.87			
*Peptococcus*	MR Egger	5.96	0.88	0.01	0.88	0.94
	IVW	5.99	0.92			
*Prevotella7*	MR Egger	18.61	0.03	0.02	0.87	0.06
	IVW	18.67	0.04			
*Ruminiclostridium-6*	MR Egger	8.91	0.71	0.02	0.65	0.80
	IVW	9.13	0.76			
*RuminococcaceaeUCG003*	MR Egger	9.42	0.31	− 0.06	0.29	0.31
	IVW	10.94	0.28			

*IDB* inflammatory disorders of the breast, *GM* gut microbiota, *IVW* inverse-variance weighted, *MR* Mendelian randomization

**Fig. 2:**
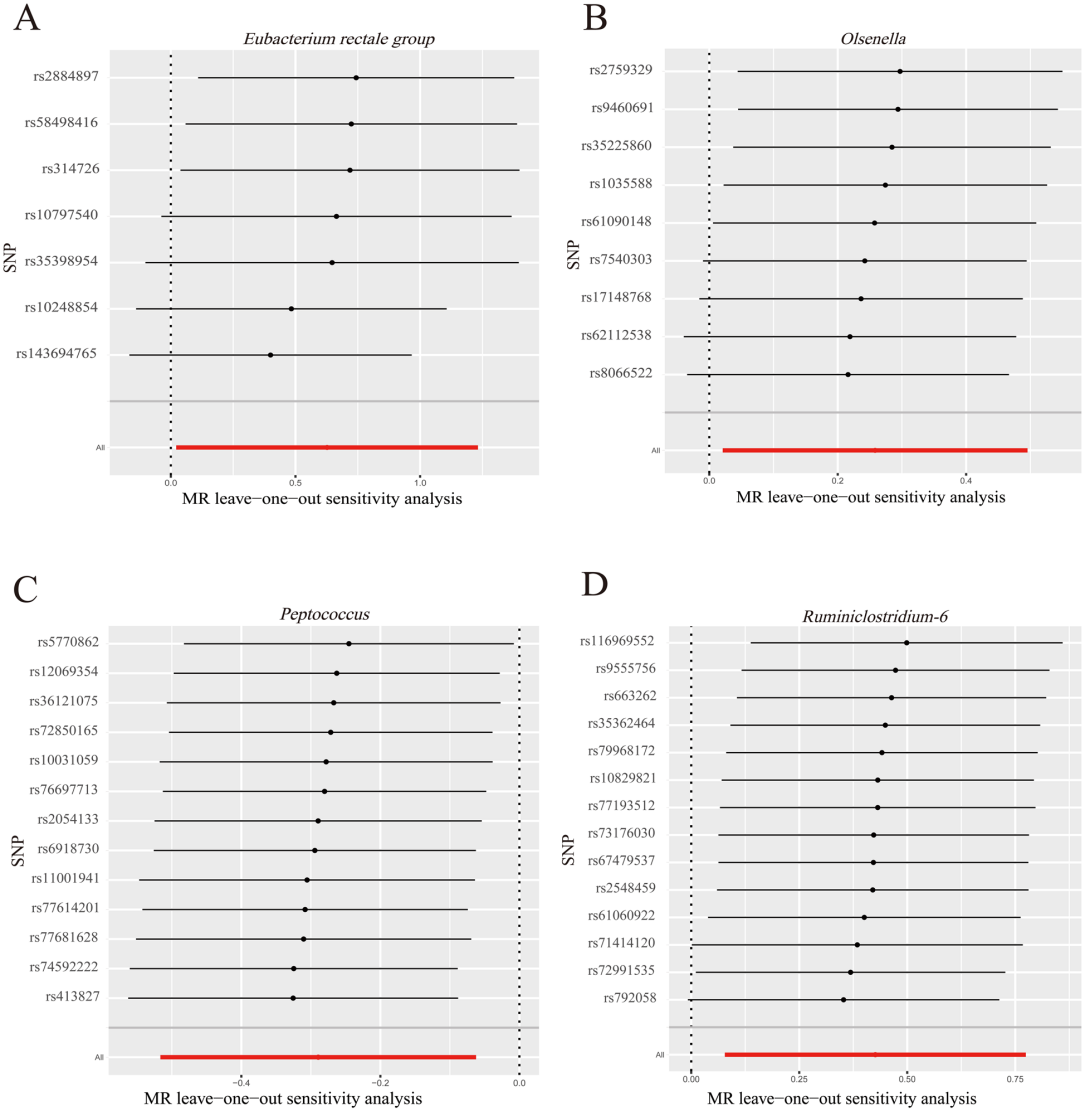
Leave-one-out plots for the causal effects between GM and IDB. **A** Leave-one-out sensitivity analysis of MR for the effect of the genus *Eubacterium rectale group* on IDB; **B** leave-one-out sensitivity analysis of MR for the effect of the genus *Olsenella* on IDB; **C** leave-one-out sensitivity analysis of MR for the effect of the genus *Peptococcus* on IDB; **D** leave-one-out sensitivity analysis of MR for the effect of the genus *Ruminiclostridium-6* on IDB. The red and black dot or bar indicated the causal estimate between GM and IDB

**Fig. 3:**
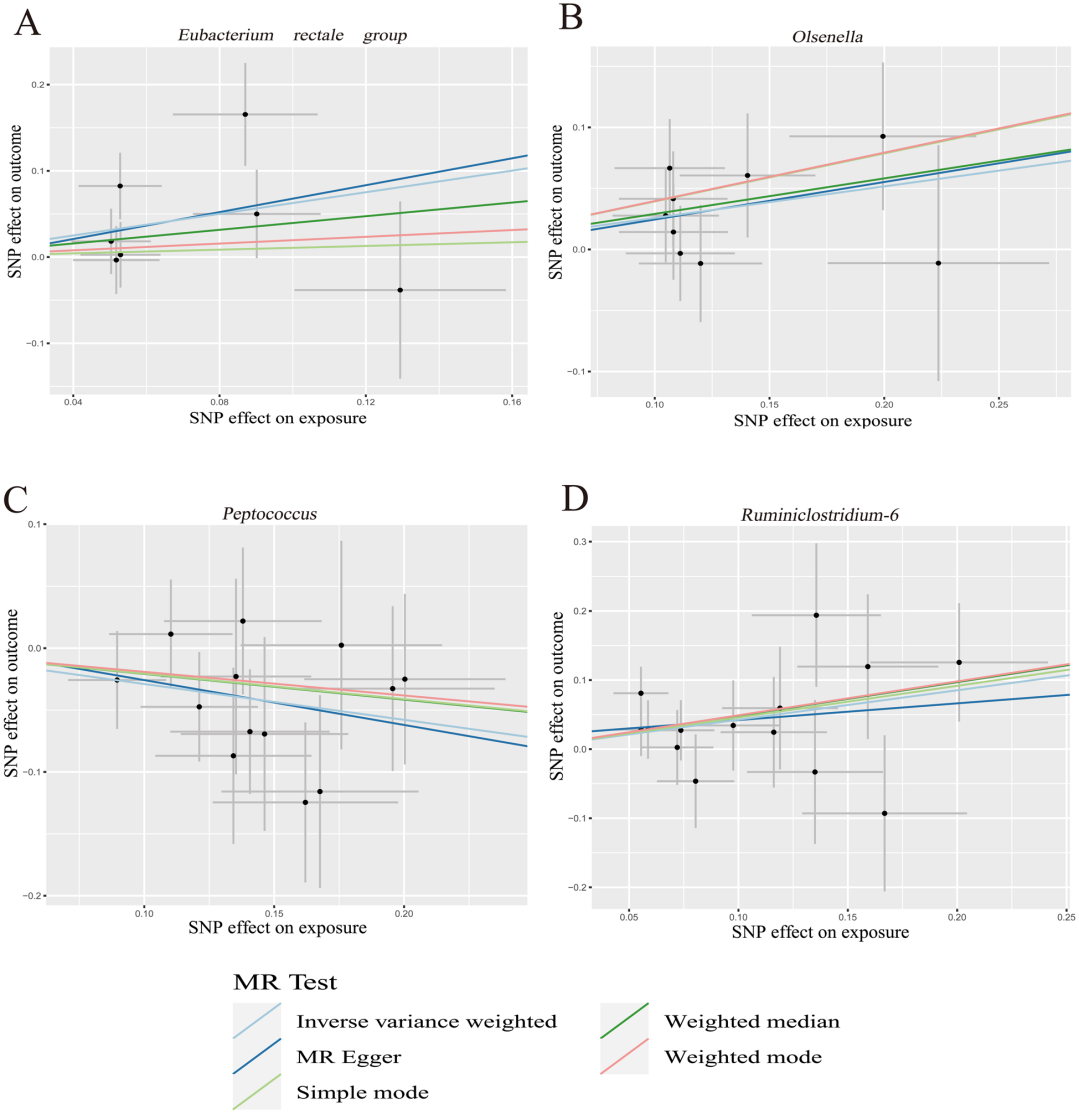
Scatter plots for the causal effects between GM and IDB. **A** The causal effect of the genus *Eubacterium rectale group* on IDB; **B** the causal effect of the genus *Olsenella* on IDB; **C** the causal effect of the genus *Peptococcus* on IDB; **D** the causal effect of the genus *Ruminiclostridium-6* on IDB. The slopes of line represented the causal effect of each method, respectively. The black dot indicated each related SNP. A negative correlation line with a slope less than 0, indicating the protective effect of GM on IDB. A positive correlation line with a slope greater than 0, indicating the anti-protective effect of GM on IDB

The leave-one-out plots (Fig. 2) and the scatter plots (Fig. 3) have shown the possible presence of potential outliers. In order to pursue the robustness of MR-Egger regression results, the method of MR-PRESSO method was used. The results were optimistic as no significant outliers were found (all *p* > 0.05, Table 2).

Finally, the main point is that the outcomes of IVW were assured after checking heterogeneity and pleiotropy. Therefore, *Eubacterium rectale group, Olsenella, Peptococcus* and *Ruminiclostridium-6* were causally related to IDB.

## Discussion

As far as we know, our study takes the lead in assessing the causality between GM and IDB in terms of the genetic level. In this study, two-sample MR analysis based on the largest GWAS data set gave fairly strong evidence that gut microbiome plays non-negligible role in the occurrence and progression of IDB, in which, metabolites may be involved in. Results displayed that *Eubacterium rectale group*, *Olsenella* and *Ruminiclostridium-6* had an anti-protective effect on IDB, whereas *Peptococcus* had a protective effect on IDB.

Several studies have reported the association between *Ruminiclostridium-6* and other disorders, although the relationship between *Ruminiclostridium-6* and IDB has not been explored. Previous studies revealed that *Ruminiclostridium-6* acted as a vital regulatory effect in colitis. *Ruminiclostridium-6* could contribute to the release of proinflammatory factors such as IL-6, IL-1β, TNF-α and IL-8 and deteriorate colitis [54]. In addition, a cohort study has shown the *Ruminiclostridium-6* was significantly enriched in community-acquired pneumonia patients, implying its potential pathogenicity [55]. IDB is an infection of mammary gland [56] that may be due to a severe disruption of the blood–milk barrier [57] caused by harmful factors (e.g., enteropathogenic bacteria), which in turn is transferred from the intestine to the breast. Current evidence focuses on the pathogenesis of rumen-induced IDB. Rumen-derived LPS decreased the expression of tight junctional proteins, in turn disrupts the blood–milk barrier and increasing permeability. Therefore, we hypothesized that *Ruminiclostridium-6* may have a performance impact on IDB via regulating proinflammatory factors to disrupt the blood–milk barrier and deteriorate IDB.

Conclusive evidence also needed to confirm how *Eubacterium rectale group* and *Olsenella* increase the risk of IDB. Although *Eubacterium rectale group* as one of butyrate-producing flora benefits to certain disorder [58], butyrate is also reported to promote tumorigenesis [59]. The evidence against *Eubacterium rectale group* have been documented. Islam et al has found *Eubacterium rectale group* inhibited CD83 to keep mice in systemic inflammation [60]. Wang et al. also revealed the *Eubacterium rectale group* played proinflammatory role in colorectal cancer [61]. Therefore, we could infer a conclusion that *Eubacterium rectale group* exacerbates IDB through systemic inflammation. For *Olsenella*, only observational study has reported its changes with disease [62, 63]. Our study verified the potential harmfulness of *Olsenella* in humans at the first time and *Olsenella* has the potential to be a candidate of biomarker of IDB.

Trillions of symbiotic GM on the surface of the human gastrointestinal mucosa maintain the host health. As the degree of IDB increased, short chain fatty acids (SCFAs) were significantly decreased [64]. A strategy of probiotics treatment may reduce the risk [65]. *Peptococcus* has a solid positive correlation with valeric acid and butyrate [66–68]. Probiotics and SCFAs may inhibit inflammation and maintain blood–milk barrier function. Research revealed SCFAs participated in the energy supply of tight junction proteins [69], suggesting its function in the developing of blood–milk barrier. Propionate acid shielded lactating women from IDB by modulating the blood–milk barrier [70]. The research also pointed that butyrate, one of SCFAs, was at dominance of modulating the inflammatory response [18, 71]. Moreover, butyrate repairs blood–milk barrier by improving tight junction proteins [72]. Although few reports concentrated on *Peptococcus* acting as a probiotic in the past, our study has found *Peptococcus* may become a candidate of probiotics therapy today. Nevertheless, more RCTs are needed to conduct to support the novel treatment.

This research has several advantages. Genetic variation is not affected by confounding factors. Thus, the measurement error between genetic variation and its effects is relatively small. Based on this, we employed MR analysis to determine the causal effect between GM and IDB. Genetic data were adopted from the latest large GWAS, keeping the robustness of IVs in the MR analysis. Several statistical techniques were performed to detect the precision of results. A two-sample MR design widely used because it avoids bias by nonoverlapping data.

However, several limitations in this study deserve noting. Firstly, weak instrumental bias may not be avoided even if satisfying the MR assumptions (IVs are closely correlated with GM taxa). Secondly, the GWAS recruited subjects only of particular race or nationality, the generalization of findings in our research could not be suitable. MR studies of cross racial may consider for better generalizability. Thirdly, MR analysis typically reveals a lifetime exposure, the existence of canalization may cause overestimation of effect size. Further RCTs should be performed to exam the effect. Fourthly, we conducted MR analysis on five species level, however, we only found eligible SNPs on genus level, thus we could try our best to enlarge the sample size to improve the effectiveness of samples. Finally, the research of biological mechanisms should be paid attention to interpret MR results.

## Conclusions

In summary, we revealed causality between IDB and GM taxa, exactly including *Ruminiclostridium-6*, *Eubacterium rectale group*, *Olsenella* and *Peptococcus*. These genera may become novel biomarkers and supply new viewpoint for probiotic treatment. However, these findings warrant further testing owing to the insufficient evidences.

### Supplementary Information

**Additional file 1: Table S1.** IVs used in MR analysis of the association between GM and IDB. **Table S2.** Casual effects of MR analysis between GM and IDB. **Table S3.** *F*-statistic results of MR analysis. **Table S4.** Results of sensitivity analysis.

## Data Availability

The GWAS data on GM originated from MiBioGen consortium, https://mibiogen.gcc.rug.nl/ [29-31] and the GWAS data relating IDB were mainly conducted by the Finngen consortium, https://r8.finngen.fi/ [32, 33].

## Abbreviations

IDB: Inflammatory disorders of the breast
LM: Lactational mastitis
NLM: Non-lactational mastitis
GM: Gut microbiota
MR: Mendelian randomization
GWAS: Genome-wide association studies
SNPs: Single nucleotide polymorphisms
IVW: Inverse variance weighted
FMT: Feces microbiota transplantation
RCTs: Randomized controlled trials
IVs: Instrumental variables
SCFAs: Short chain fatty acid

## References

[1] Scott DM (2022). Inflammatory diseases of the breast. Best Pract Res Clin Obstet Gynaecol.

[2] Lai BY, Yu BW, Chu AJ (2021). Risk factors for lactation mastitis in China: a systematic review and meta-analysis. PLoS ONE.

[3] Kamal RM, Hamed ST, Salem DS (2009). Classification of inflammatory breast disorders and step by step diagnosis. Breast J.

[4] Blackmon MM, Nguyen H, Mukherji P (2023). Acute mastitis.

[5] Tsai MJ, Huang WC, Wang JT (2020). Factors associated with treatment duration and recurrence rate of complicated mastitis. J Microbiol Immunol Infect.

[6] Kataria N, Lam DL, Parker EU (2021). Radiology-pathology correlation: inflammatory conditions of the breast. Curr Breast Cancer Rep.

[7] Gopalakrishnan Nair C, Hiran JP (2015). Inflammatory diseases of the non-lactating female breasts. Int J Surg.

[8] Shi L, Wu J, Hu Y (2022). Biomedical indicators of patients with non-puerperal mastitis: a retrospective study. Nutrients.

[9] Pevzner M, Dahan A (2020). Mastitis while breastfeeding: prevention, the importance of proper treatment, and potential complications. J Clin Med.

[10] Jahanfar S, Ng CJ, Teng CL (2016). Antibiotics for mastitis in breastfeeding women. Sao Paulo Med J.

[11] Anderson PO (2022). Guidelines for reporting cases of medication use during lactation. Breastfeed Med.

[12] Costa Morais Oliveira V, Cubas-Vega N, López Del-Tejo P (2021). Non-lactational infectious mastitis in the Americas: a systematic review. Front Med (Lausanne)..

[13] Patel SH, Vaidya YH, Patel RJ (2017). Culture independent assessment of human milk microbial community in lactational mastitis. Sci Rep.

[14] Yue Q, Cai M, Xiao B (2022). The microbiota-gut-brain axis and epilepsy. Cell Mol Neurobiol.

[15] Hu X, He Z, Zhao C (2023). Gut/rumen-mammary gland axis in mastitis: gut/rumen microbiota-mediated "gastroenterogenic mastitis". J Adv Res.

[16] Rodríguez JM, Fernández L, Verhasselt V (2021). The gut-breast axis: programming health for life. Nutrients.

[17] Zhao C, Hu X, Bao L (2021). Aryl hydrocarbon receptor activation by *Lactobacillus reuteri* tryptophan metabolism alleviates *Escherichia coli*-induced mastitis in mice. PLoS Pathog.

[18] Hu X, Guo J, Zhao C (2020). The gut microbiota contributes to the development of *Staphylococcus aureus*-induced mastitis in mice. ISME J.

[19] Ma C, Sun Z, Zeng B (2018). Cow-to-mouse fecal transplantations suggest intestinal microbiome as one cause of mastitis. Microbiome.

[20] Crepinsek MA, Taylor EA, Michener K (2020). Interventions for preventing mastitis after childbirth. Cochrane Database Syst Rev.

[21] Margolis KG, Cryan JF, Mayer EA (2021). The microbiota-gut-brain axis: from motility to mood. Gastroenterology.

[22] Rinninella E, Raoul P, Cintoni M (2019). What is the healthy gut microbiota composition? A changing ecosystem across age, environment, diet, and diseases. Microorganisms..

[23] Wang YZ, Shen HB (2020). Challenges and factors that influencing causal inference and interpretation, based on Mendelian randomization studies. Zhonghua Liu Xing Bing Xue Za Zhi.

[24] Zheng J, Baird D, Borges MC (2017). Recent developments in Mendelian randomization studies. Curr Epidemiol Rep.

[25] Long Y, Tang L, Zhou Y (2023). Causal relationship between gut microbiota and cancers: a two-sample Mendelian randomisation study. BMC Med.

[26] Zhang Y, Zhang X, Chen D (2022). Causal associations between gut microbiome and cardiovascular disease: a Mendelian randomization study. Front Cardiovasc Med.

[27] Chen M, Xie CR, Shi YZ (2022). Gut microbiota and major depressive disorder: a bidirectional Mendelian randomization. J Affect Disord.

[28] de Leeuw CA, Savage JE, Bucur IG (2021). Understanding the assumptions underlying Mendelian randomization. Eur J Hum Genet.

[29] Kurilshikov A, Medina-Gomez C, Bacigalupe R (2021). Large-scale association analyses identify host factors influencing human gut microbiome composition. Nat Genet.

[30] Wray NR, Ripke S, Mattheisen M (2018). Genome-wide association analyses identify 44 risk variants and refine the genetic architecture of major depression. Nat Genet.

[31] CONSORTIUM M. MiBioGen. https://mibiogen.gcc.rug.nl/. Accessed 16 Mar 2023.

[32] Kurki MI KJ, Palta P, Sipilä TP, Kristiansson K, Donner K, et al. . FinnGen: unique genetic insights from combining isolated population and national health register data, 2022.03.03.22271360.

[33] FINNGEN. FinnGen R8 release. https://r8.finngen.fi/. Accessed 22 Mar 2023.

[34] Wang J, Kurilshikov A, Radjabzadeh D (2018). Meta-analysis of human genome-microbiome association studies: the MiBioGen consortium initiative. Microbiome.

[35] Angelopoulou A, Field D, Ryan CA (2018). The microbiology and treatment of human mastitis. Med Microbiol Immunol.

[36] Kornfeld HW, Mitchell KB (2021). Management of idiopathic granulomatous mastitis in lactation: case report and review of the literature. Int Breastfeed J.

[37] Li P, Wang H, Guo L (2022). Association between gut microbiota and preeclampsia-eclampsia: a two-sample Mendelian randomization study. BMC Med.

[38] Liu K, Zou J, Fan H (2022). Causal effects of gut microbiota on diabetic retinopathy: a Mendelian randomization study. Front Immunol.

[39] Sanna S, van Zuydam NR, Mahajan A (2019). Causal relationships among the gut microbiome, short-chain fatty acids and metabolic diseases. Nat Genet.

[40] Jia J, Dou P, Gao M (2019). Assessment of causal direction between gut microbiota-dependent metabolites and cardiometabolic health: a bidirectional mendelian randomization analysis. Diabetes.

[41] Burgess S, Dudbridge F, Thompson SG (2016). Combining information on multiple instrumental variables in Mendelian randomization: comparison of allele score and summarized data methods. Stat Med.

[42] Bowden J, Davey Smith G, Burgess S (2015). Mendelian randomization with invalid instruments: effect estimation and bias detection through Egger regression. Int J Epidemiol.

[43] Hartwig FP, Davey Smith G, Bowden J (2017). Robust inference in summary data Mendelian randomization via the zero modal pleiotropy assumption. Int J Epidemiol.

[44] Verbanck M, Chen CY, Neale B (2018). Detection of widespread horizontal pleiotropy in causal relationships inferred from Mendelian randomization between complex traits and diseases. Nat Genet.

[45] Lin L, Luo P, Yang M (2022). Causal relationship between osteoporosis and osteoarthritis: a two-sample Mendelian randomized study. Front Endocrinol (Lausanne).

[46] Gao Y, Fan ZR, Shi FY (2023). Hypothyroidism and rheumatoid arthritis: a two-sample Mendelian randomization study. Front Endocrinol (Lausanne).

[47] He J, Luo X, Xin H (2022). The effects of fatty acids on inflammatory bowel disease: a two-sample Mendelian randomization study. Nutrients.

[48] Xu J, Zhang S, Tian Y (2022). Genetic causal association between iron status and osteoarthritis: a two-sample mendelian randomization. Nutrients.

[49] Levin MG, Judy R, Gill D (2020). Genetics of height and risk of atrial fibrillation: a Mendelian randomization study. PLoS Med.

[50] Shi J, Tian J, Fan Y (2022). Intelligence, education level, and risk of Parkinson's disease in European populations: a Mendelian randomization study. Front Genet.

[51] Staiger D, Stock JH (1997). Instrumental variables regression with weak instruments. Econometrica.

[52] Yu XH, Yang YQ, Cao RR (2021). The causal role of gut microbiota in development of osteoarthritis. Osteoarthr Cartil.

[53] Liu B, Ye D, Yang H (2022). Two-sample Mendelian randomization analysis investigates causal associations between gut microbial genera and inflammatory bowel disease, and specificity causal associations in ulcerative colitis or Crohn's disease. Front Immunol.

[54] Ge H, Cai Z, Chai J (2021). Egg white peptides ameliorate dextran sulfate sodium-induced acute colitis symptoms by inhibiting the production of pro-inflammatory cytokines and modulation of gut microbiota composition. Food Chem.

[55] Xiao Q, Tan S, Liu C (2023). Characterization of the microbiome and host's metabolites of the lower respiratory tract during acute community-acquired pneumonia identifies potential novel markers. Infect Drug Resist.

[56] Omranipour R, Vasigh M (2020). Mastitis, breast abscess, and granulomatous mastitis. Adv Exp Med Biol.

[57] Wall SK, Hernández-Castellano LE, Ahmadpour A (2016). Differential glucocorticoid-induced closure of the blood-milk barrier during lipopolysaccharide- and lipoteichoic acid-induced mastitis in dairy cows. J Dairy Sci.

[58] Lu H, Xu X, Fu D (2022). Butyrate-producing Eubacterium rectale suppresses lymphomagenesis by alleviating the TNF-induced TLR4/MyD88/NF-κB axis. Cell Host Microbe.

[59] Okumura S, Konishi Y, Narukawa M (2021). Gut bacteria identified in colorectal cancer patients promote tumourigenesis via butyrate secretion. Nat Commun.

[60] Islam SMS, Ryu HM, Sayeed HM (2021). Eubacterium rectale attenuates HSV-1 induced systemic inflammation in mice by inhibiting CD83. Front Immunol.

[61] Wang Y, Wan X, Wu X (2021). Eubacterium rectale contributes to colorectal cancer initiation via promoting colitis. Gut Pathog.

[62] Kesim B, Ülger ST, Aslan G (2023). Amplicon-based next-generation sequencing for comparative analysis of root canal microbiome of teeth with primary and persistent/secondary endodontic infections. Clin Oral Investig.

[63] Zmysłowska-Polakowska E, Płoszaj T, Skoczylas S (2023). Evaluation of the oral bacterial genome and metabolites in patients with Wolfram syndrome. Int J Mol Sci.

[64] Wang Y, Nan X, Zhao Y (2021). Rumen microbiome structure and metabolites activity in dairy cows with clinical and subclinical mastitis. J Anim Sci Biotechnol.

[65] Barker M, Adelson P, Peters MDJ (2020). Probiotics and human lactational mastitis: a scoping review. Women Birth.

[66] Liu S, Fan Z (2022). Effects of dietary protein restriction on colonic microbiota of finishing pigs. Animals (Basel)..

[67] Li X, Xie Q, Huang S (2021). Digestion and fermentation characteristics of sulfated polysaccharides from Gracilaria chouae using two extraction methods in vitro and in vivo. Food Res Int.

[68] Sandri M, Dal Monego S, Conte G (2017). Raw meat based diet influences faecal microbiome and end products of fermentation in healthy dogs. BMC Vet Res.

[69] Liu Q, Liu J, Roschmann KIL (2013). Histone deacetylase inhibitors up-regulate LL-37 expression independent of toll-like receptor mediated signalling in airway epithelial cells. J Inflamm (Lond).

[70] Wang J, Wei Z, Zhang X (2017). Propionate protects against lipopolysaccharide-induced mastitis in mice by restoring blood-milk barrier disruption and suppressing inflammatory response. Front Immunol.

[71] Wang JJ, Wei ZK, Zhang X (2017). Butyrate protects against disruption of the blood-milk barrier and moderates inflammatory responses in a model of mastitis induced by lipopolysaccharide. Br J Pharmacol.

[72] Zhao C, Bao L, Qiu M (2022). Commensal cow Roseburia reduces gut-dysbiosis-induced mastitis through inhibiting bacterial translocation by producing butyrate in mice. Cell Rep.

